# Genetic Diversity and Evolution of Chinese Traditional Medicinal Fungus *Polyporus umbellatus* (Polyporales, Basidiomycota)

**DOI:** 10.1371/journal.pone.0058807

**Published:** 2013-03-15

**Authors:** Xiaoke Xing, Xueting Ma, Miranda M. Hart, Airong Wang, Shunxing Guo

**Affiliations:** 1 Institute of Medicinal Plant Development, Chinese Academy of Medical Science and Peking Union Medical College, Beijing, China; 2 Biology, University of British Columbia Okanagan, Kelowna, Canada; Beijing Institute of Genomics, Chinese Academy of Sciences, China

## Abstract

**Background:**

*Polyporus umbellatus* is an important medicinal fungus distributed throughout most area of China. Its wide distribution may have resulted in substantial intraspecific genetic diversity for the fungus, potentially creating variation in its medical value. To date, we know little about the intraspecific genetic diversity of *P. umbellatus*.

**Methodology/Principal Findings:**

The objective of this research was to assess genetic differences of *P. umbellatus* from geographically diverse regions of China based on nrDNA ITS and 28S rRNA (LSU, large subunit) sequences. Significant sequence variations in the ITS and LSU sequences were detected. All sclerotial samples were clustered into four clades based on phylogenetic analysis of ITS, LSU and a combined data set of both regions. Heterogeneity of ITS and LSU sequences was detected in 5 and 7 samples respectively. All clone sequences clustered into the same clade except for one LSU clone sequences (from Henan province) which clustered into two clades (Clade I and Clade II). Significant genetic divergence in *P. umbellatus* was observed and the genetic diversification was greater among sclerotial samples from Shaanxi, Henan and Gansu provinces than among other provinces. Polymorphism of ITS and LSU sequences indicated that in China, *P. umbellatus* may spread from a center (Shaanxi, Henan and Gansu province) to other regions.

**Conclusions/Significance:**

We found sclerotial samples of *P. umbellatus* contained levels of intraspecific genetic diversity. These findings suggested that *P. umbellatus* populations in Shaanxi, Henan and Gansu are important resources of genetic diversity and should be conserved accordingly.

## Introduction

*Polyporus umbellatus* (Pers.) Fr. (also known as *Grifola umbellata* (Pers.) Pilát and *Dendropolyporus umbellatus* (Pers.) Jülich), is a widespread Basidiomycota fungus which occurs in both broadleaf and coniferous forests. *P. umbellatus* has a distribution primarily in China, Japan, Korea and other temperate regions of the Northern Hemisphere [1]–[4]. In China, *P. umbellatus* has a wide distribution in most provinces [5]. The fruiting body of *P. umbellatus* is edible [6], while the sclerotium is of medicinal value [2], [7]. The sclerotium of *P. umbellatus* can survive in soils for long periods of time, and produce new sclerotia directly from the older ones under appropriate conditions [5]. The sclerotia have been used in traditional Chinese medicine for centuries and are used to treat edema and promote diuretic processes [8]. In recent years, a polysaccharide from *P. umbellatus* sclerotia was reported to promote anti-tumor and immunomodulating activities [9], [10].

Presently, wild sclerotia are the main source of medicinal material. The lack of effective protection along with increasing commercial demand has led to excessive harvest and a dramatic decline of wild resources in China [11]. Though there have been attempts to mass produce of *P. umbellatus* in some Chinese provinces (such as Shanxi and Shaanxi) [12], there have been problems with both the quality of the sclerotia and production efficiency.

Morphologically, sclerotia of *P. umbellatus* are divided into two groups, *Ji Shi Ling* (sclerotia with a slim body and more branches) and *Zhu Shi Ling* (sclerotia with a thick body and fewer branches) [5]. Until now, this was the only indication of intraspecific difference within *P. umbellatus*. Liu et al. [13] compared the rDNA sequences of the two kinds of sclerotia and found no sequence differences between them. However, this study compared sclerotia from a single geographic region (Qinba Mountain, China).

*Polyporus* is the type genus of the family Polyporaceae and is a cosmopolitan genus including ecologically diverse species [14], [15]. Continental phylogeographic structure is common in basidiomycete macrofungi [16] but we know little about this for *Polyporus*, despite its economic and ecological importance. In recent years, molecular tools have been employed to systematically characterize other *Polyporus* species [15], [17], [18]. However, the classification of *P. umbellatus* remains ambiguous. *P. umbellatus* has unique basidiocarps (connate, centrally stipitate basidiocarps arising from sclerotia) and is considered to be a phylogenetically distinct lineage from other species [15]. Sotome et al. [15] confirmed *Polyporus* as a polyphyletic genus and recognized six clades containing species of *Polyporus* and other allied genera. *P. umbellatus* was not included in any major clades and its phylogenetic placement was not clarified.

Commercial production of sclerotia is currently thwarted by lack of information about the genetic structure of *P. umbellatus* and a lack of knowledge about the biogeographic structure of *P. umbellatus* germplasm. To date, intraspecific genetic diversity of *P. umbellatus* has never been studied. The wide spread distribution of *P. umbellatus* in China provides an unrivaled opportunity to test for genetic differences among sclerotia from different geographic sites. We have collected sclerotia of *P. umbellatus* from twelve provinces throughout China. The purpose of this study was to evaluate the intraspecific genetic variation occurring among *P. umbellatus* from different geographic sites in China based on rDNA internal transcribed spacer (ITS 1 and ITS 2 regions) and LSU (28S rRNA) sequences. Furthermore, we explored the possible origin and biogeography of *P. umbellatus*. Examining the genetic diversity and biogeography of *P. umbellatus* will provide insight into its origins and as well as mechanisms of macro-fungal dispersal. It will also provide valuable information for the protection and sustainability of this rare natural resource.

## Materials and Methods

### Ethics Statement

No specific permits were required for the described field studies.

### Sclerotial samples and morphological classification

Forty-two samples of fresh sclerotia were collected from the following twelve provinces: Gansu, Qinghai, Shaanxi, Shanxi, Henan, Hebei, Sichuan, Yunnan, Heilongjiang, Jilin, Liaoning and Beijing (for site information see Table S1). For each sample, at least eight sclerotia from different individuals growing 30–50 m apart were chosen. In total, more than 336 individual sclerotia were collected. Once the fresh sclerotia were collected, they were delivered to the lab within few days for further processing. Sclerotia from each sample were deposited in a herbarium (Institute of Medicinal Plant Development, Chinese Academy of Medical Sciences). According to morphological classification of sclerotia [5], most of the sclerotial samples were *Zhu Shi Ling* except for samples from Hanyin, Shaanxi Province and from Northeast China (Jilin, Liaoning and Heilongjiang), which were *Ji Shi Ling*.

### DNA extraction

For each sample, five different sclerotia were randomly selected for DNA extraction. In total, 210 DNA extractions were made. The sclerotia were first surface sterilized by dipping in 75% ethanol for 1 min, followed by a solution of 3.5% (v/v) Chlorox for 2 min, and finally 75% ethanol for 30 s. Sterilized sclerotia were washed with sterile distilled water three times and blotted with bibulous paper. The sclerotia were then cut in half and 100 mg of medullar tissue was removed and ground with a mortar and pestle in liquid nitrogen. Genomic DNA was extracted using the E.Z.N.A. ™Fungal DNA kit (Omega) following the manufacturer's instructions.

### PCR amplification and sequencing

Sequences of rDNA have often been used for phylogenetic analyses and evolutionary relationships between taxa because they are well-characterized and easily accessible [19]. The ITS-region, 28S gene (LSU) or a combination of both these regions has been used to infer phylogenetic relationships for various species of Polyporales and other basidiomycete groups [15], [18], [20], [21]. In this study, we amplified the internal transcribed spacer region 1, the 5.8S rDNA gene and the ITS region 2 using the universal primer pair ITS1 and ITS4 [22]. Amplification was performed in a 50 µl reaction mixture containing 10 mM Tris-HCl (pH 9.0), 50 mM KCl, 0.1% Trition X 100, 2 mM MgCl_2_, 0.2 mM dNTPs, 0.4 µM primers, 0.5 unit of Taq polymerase, and 2 µl of fungal genomic DNA. The thermal cycling program was as follows: initial denaturation 94°C for 2 min, followed by 35 cycles: 94°C for 1 min, 55°C for 30 s and 72°C for 45 s, and 10 min at 72°C for a final extension. PCR primers LROR and LR7 [23] were used to amplify the LSU. The protocol was the same as described above, except that the extension time was extended to 2 min. Amplification products were electrophoresed on a 1.0% agarose gel and checked to ensure that a single DNA band was produced of the expected size (∼600 bp and ∼1400 bp for ITS and LSU PCR products, respectively). For sequencing, a QiaQuick PCR purification kit (Qiagen, Hilden, Germany) was used to purify PCR products from unincorporated nucleotides, excess primer and salts as well as primer dimers. Sequencing reactions were performed on an ABI-310 capillary sequencer using an ABI dye-terminator kit (ABI/Perkin-Elmer) following the protocol supplied by the manufacturer.

### Sequence alignment and phylogenetic analyses

Sequences of ITS and LSU were aligned with Clustal X version 2.0 [24], and ambiguous regions in both sides of each region were excluded. For ITS sequences, the subsequences including partial 18S (7 bp), ITS1 (184–212 bp), 5.8S (161 bp), ITS2 (178–203 bp), and partial 28S (40 bp) were used. Pairwise distance matrices and minimum evolutionary (ME) phylogenetic analysis were conducted with Kimura 2-Parameter model using MEGA version 5 [25]. Bootstrap analyses were performed for 1000 replicates. For Maximum Parsimony (MP) analyses, an initial heuristic search of 100 random taxon addition replicate searches was conducted with TBR branch-swapping, MAXTREES set to autoincrease, without constraints, all characters unordered and equally weighted, gaps treated as missing data, and with two trees held during each stepwise addition cycle. The shortest trees from this analysis were used as starting trees in a second heuristic search, with the same parameters described above, to find the most parsimonious trees. For the Maximum Likelihood (ML) analyses, jModelTest 2 [26] was used to identify the model of DNA substitution that best fits. HKY+G was estimated as the best-fit likelihood model for the combined ITS and LSU datasets. The best-fit likelihood model for ITS and LSU was K80+I and HKY respectively. Maximum likelihood analysis was performed with PAUP 4.0b10 [27]. Genetic parameters of nucleotide diversity were calculated by DnaSP software (version 5.10.01) [28]. Genetic differentiation between populations and analysis of molecular variance (AMOVA) were assessed using the Program Arlequin 3.5 [29]. For AMOVA, *F*_SC_ estimates the variation among populations relative to a regional grouping of populations. *F*_ST_ estimates the proportions of genetic variation within populations relative to the genetic variation for the whole samples. *F*_CT_ estimates the proportion of genetic variation among groups of populations relative to the whole species. Samples from the same province were regarded as one population. The one sample from Beijing (ZL-BJ-1) was put into the population of Hebei for the following analyses because location of Beijing is geographically located in the Hebei Province.

### Single Strand Conformation Polymorphis (SSCP) analysis

In general, both ITS and LSU sequences of the five individual sclerotia from one sample do not differ, except for individual sclerotia from nine samples that exhibited heterogeneous peaks in direct sequencing. For better understanding of the heterogeneity within ITS and LSU of these nine samples, single strand conformation polymorphism (SSCP) was used in this study. SSCP is a fragment-based approach which offers a convenient method for detecting sequence diversity among many individuals, allowing for selection of an informative subset of individuals for downstream analysis [30]. Based on ITS and LSU sequences, sclerotial samples exhibiting heterogeneous peaks in direct sequencing were selected for SSCP analyses. ITS sequences and LSU sequences were amplified with the same primers and PCR conditions as described previously. The PCR products were purified using the QiAquick PCR purification kit (Qiagen) and cloned using the pGEM-T vector (TakaRa, Japan) and *Escherochia coli* DH5α. SSCP screening of colony PCR products was conducted at 10°C using the DCodeTM Universal Mutation Detection System (Bio-Rad, Hercules, CA). PCR products (3 µl) were mixed with the same volume of denaturing buffer (95% formamide, 20 mM EDTA, 0.05% bromophenol blue, 0.05% xylene cyanol). The mixtures were heated at 95°C for 10 min and then immediately cooled on ice for 15 min. Denatured PCR products were then loaded into the slots of a polyacrylamide gel composed of 8% acrylamidebisacrylamide (37.5∶1), 1×TBE buffer, 5% glycerol, 0.1% ammonium persulfate, and 0.1% tetramethylethylenediamine (TEMED). After running for 5 min at 300 V, electrophoresis was continued for 8 h at 260 V. Finally, the gel was silver-stained [31]. Different migration profiles between clones were compared. Representative clones with distinct patterns were selected for sequencing with the universal primer M13-47.

## Results

### Sequence variations of nrDNA ITS and LSU genes among sclerotia of P. umbellatus

We looked at the rDNA from two regions to assess whether populations of *P. umbellatus* from throughout China had distinct genetic biodiversity. The ITS sequences ranged from 622–664 bp with the shortest and longest found in samples from Hanyin Shaanxi and Huzhu Qinghai respectively. We found nucleotide variations at 148 sites, including 93 sites with alignment gaps or missing data (79 parsimony informative sites and 14 singleton variable sites), 49 parsimony informative sites and 6 singleton variable sites. The 20 singleton sites (6+14 from above) were excluded from further analysis for one polymorphism at such a site was represented by only one sample. Most of 128 parsimony informative sites (79+49 from above) were located in ITS 1 (69 sites) and ITS2 regions (56 sites), and the other 3 located in 28S regions. Detailed information of base transition in these parsimony informative sites is listed in Table S2.

For the 1413–1423 bp LSU sequences, the shortest sequence was found in Luanchuan, Henan Province (ZL-HN-4 and ZL-HN-5), and the longest sequence was found in Hanyin and Liuba, Shaanxi Province (ZL-ShX-5, ZL-ShX-6, ZL-ShX-7 and ZL-ShX-9). Nucleotide variations occurred at 128 sites, including 78 parsimony informative sites and 50 singleton variable sites. Detailed information of base transition in these 78 parsimony informative sites is listed in Table S3. The sequences for ITS and LSU were deposited in GenBank (accession numbers HQ225479–HQ225509, JX110713–JX110771).

### Haplotypes within the sclerotial samples

**Figure 1:**
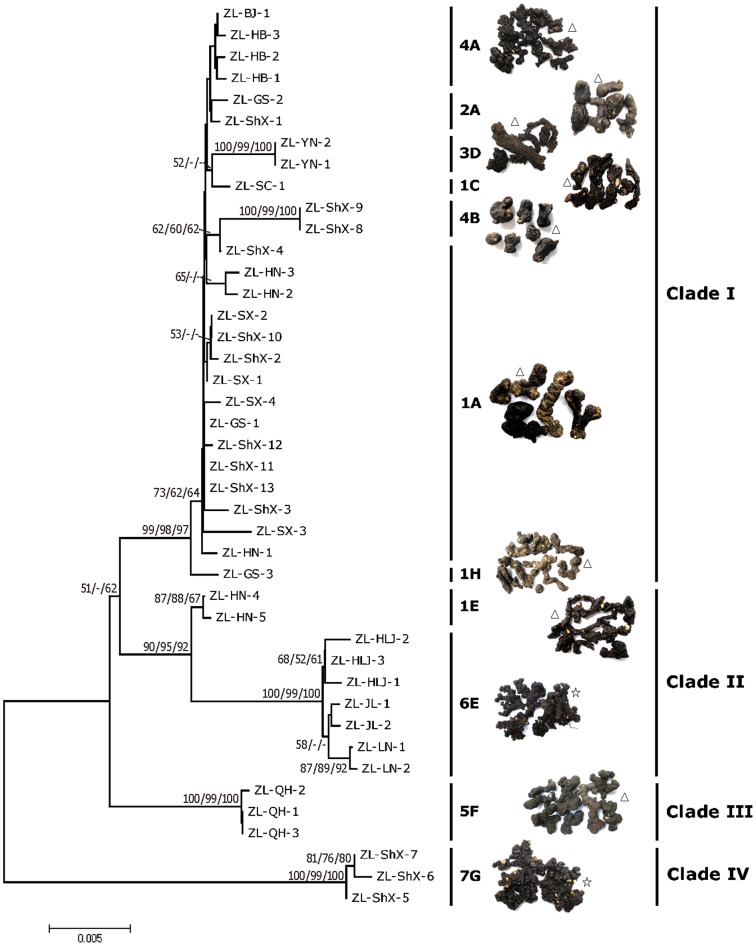
Phylogenetic analyses of 42 *Polyporus umbellatus* sclerotia based on the combined data set of nrDNA ITS and LSU (28S rRNA). Bootstrap values from minimum evolution, maximum parsimony and maximum likelihood higher than 50% are shown at the nodes. The representative sclerotial pictures of each combined haplotypes are shown aside. Arabic numbers (1–7) indicate ITS haplotypes and capital letters (A–G) indicate LSU haplotypes. Sample codes: The capital letters after ZL indicate the provinces names: ShX “Shaanxi Province, SX “Shanxi Province”, HB “Hebei Province”, HN “Henan Province”, SC “Sichuan Province”, GS “Gansu Province”, QH “Qinghai Province”, YN “Yunnan Province”, JL “Jilin Province”, LN “Liaoning Province”, HLJ “Heilongjiang Province”, BJ “Beijing”. Symbols marked at the side of sclerotial images refer to sclerotial morphotypes: ▵ “*Zhu Shi Ling*”, ??? “*Ji Shi Ling*”. Arabic numbers after province name indicate the sample number from same province (also see Table S1).

In terms of haplotypes, we found significant inter- and intra-population variation. That is, we found that sclerotial samples from the same province contained different haplotypes (i.e Shaanxi Province), but we also found one haplotype could be found in samples from different provinces (i.e. haplotype 1A, Fig. 1).

In total, seven distinct ITS haplotypes, eight distinct LSU haplotypes and eleven combined haplotypes were found within the 42 sclerotial samples used in this study (Fig. 1). For the seven ITS haplotypes (1–7), haplotype 1 was the most dominant, and was represented by 19 samples from 5 provinces (Shaanxi, Gansu, Shanxi, Henan and Sichuan). Haplotype 5 included 3 samples only from Qinghai Province. Haplotype 6 was represented by 7 samples all from Northeast China (Jilin, Liaoning and Heilongjiang). Other haplotypes were found in different provinces and representation ranged from 2 to 6 samples respectively (Table S2).

For the eight LSU Haplotypes (A–H), haplotype A was the most dominant and included 21 samples from 6 provinces (Shaanxi, Gansu, Shanxi, Henan, Hebei and Beijing). Haplotype F included 3 samples only from Qinghai Province. Haplotype E included samples mainly from Northeast China. Interestingly, two samples from Henan Province (ZL-HN-4 and ZL-HN-5) were also included in haplotype E, which were included in ITS haplotype 1. Other haplotypes included 1 to 3 samples (Table S3).

For the eleven combined haplotypes, haplotype 1A was the most dominant and included 15 samples from 4 provinces (Shaanxi, Gansu, Shanxi and Henan). Haplotype 6E included samples all from Northeast China and haplotype 5F included samples all from Qinghai Province. Other haplotypes included 1 to 4 samples (Fig. 1). For each combined haplotype, a representative picture is shown beside the haplotype number. (Fig. 1). All the combined haplotypes were *Zhu Shi Ling* except for 6E and 7G (*Ji Shi Ling*) which were collected from Hanyin, Shaanxi Province and Northeast China respectively.

### Phylogenetic analysis

Minimum evolutionary phylogenetic analyses, MP analyses and ML analyses were performed using ITS and LSU sequences, as well their combined data in order to determine the phylogenetic distance among all sclerotial samples. Similar topological structures were found in the phylogenetic trees constructed when using both ITS and LSU sequences (Fig. S1). Four clades were differentiated in the three trees, with both regions distinguishing one major clade (I) and three small clades (II, III and IV) (Fig. 1, Fig. S1). The composition of samples from each clade was almost identical in the three trees (except for the topological location changes of clade II and clade III) (Fig. S1). The parameters of nucleotide diversity and haplotype diversity were usually higher in the ITS sequences than in the LSU sequences (Table 1).

**Table 1:** Genetic diversity of *Polyporus umbellatus* collected from different provinces*.

Provinces (Populations)	No. of sequences	No. of polymorphic sites		Average no. of nucleotide differences, K		Nucleotide diversity, Pi (%)		Haplotype diversity, h	
		ITS	LSU	ITS	LSU	ITS	LSU	ITS	LSU
Shaanxi	13	47	39	16.99	13.77	2.80±0.36	0.98±0.14	0.86±0.09	0.86±0.06
Gansu	3	3	5	2.00	3.33	0.32±0.18	0.24±0.11	1.00±0.27	1.00±0.27
Henan	5	5	24	2.60	14.2	0.41±0.17	1.00±0.17	0.80±0.16	0.80±0.16
Hebei	3	2	1	1.17	0.67	0.21±0.15	0.05±0.04	1.00±0.27	0.67±0.20
Shanxi	4	8	2	4.00	1.00	0.64±0.25	0.07±0.05	0.83±0.22	0.50±0.27
Yunnan	2	0	0	0.00	0.00	0.00	0.00	0.00	0.00
Qinghai	3	1	0	0.67	0.00	0.10±0.10	0.00	0.67±0.31	0.00
Heilongjiang	3	0	5	0.00	3.33	0.00	0.24±0.11	0.00	1.00±0.27
Jilin	2	0	2	0.00	2.00	0.00	0.14±0.10	0.00	1.00±0.50
Liaoning	2	1	0	1.00	0.00	0.15±0.15	0.00	1.00±0.50	0.00
Total samples	42	68	82	12.82	14.43	2.13±0.032	1.03±0.15	0.92±0.02	0.91±0.04

* Forty-two samples were sequenced in this study (Table S1). The genetic parameters of nucleotide diversity were estimated by DnaSP software (version 5.10.01). The “polymorphic sites” include both informative sites and singleton sites.

Clade I was composed of sclerotia from most of the provinces except Qinghai province and 3 provinces of Northeast China (Jilin, Liaoning and Heilongjiang). Clade I was supported by bootstrap support values of 99%, 98%,and 97% in three phylogenetic trees, and included four ITS haplotypes, five LSU haplotypes and six combined haplotypes, respectively (Fig. 1, Fig. S1)

Three samples from Qinghai Province, ZL-QH-1, ZL-QH-2 and ZL-QH-3 clustered together as clade II in the ITS tree (bootstrap value of 99%, 99% and 100% in three phylogenetic trees) but were included in clade III using LSU and the combined trees (bootstrap values of 100%, 99% and 100% in three phylogenetic trees). This clade (Samples from Qinghai Province) only included one ITS haplotype, one LSU haplotype and one combined haplotype (Fig. 1, Fig. S1).

Seven samples from Northeast China clustered together as clade III in ITS tree (bootstrap of 100%, 99% and 100% in three phylogenetic trees), but were grouped with clade II for LSU and the combined trees (bootstrap value of 100%, 99% and 100% in three phylogenetic trees). Two samples from Henan province, HN-4 and HN-5 were also clustered in Clade II when using LSU and the combined phylogenetic trees (Fig. 1), but these clustered with Clade I using the ITS phylogenetic tree (Fig. S1).

Three samples collected from Hanyin, Shaanxi Province (ZL-ShX-5, ZL-ShX-6 and ZL-ShX-7) formed clade IV which had a distant relationship with other samples (Fig. 1, Fig. S1). This clade was robust for trees from all regions.

### Population structure

Significant genetic differentiation was detected among sclerotia samples using both ITS and LSU regions. The parameters of nucleotide diversity and haplotype diversity were usually higher in the ITS sequences than in the 28S rDNA sequences (Table 1). For ITS sequences, significant differentiation was detected for Hebei versus Shanxi, Shanxi versus Qinghai and Heilongjiang, Shaanxi versus Jilin, Henan versus Qinghai and Heilongjiang. For LSU sequences, significant differentiation was found in Hebei versus Gansu, Yunnan versus Shaanxi and Shaanxi versus Henan (Table 2). Analyses of molecular variance (AMOVA) based on haplotype frequencies showed significant F_CT_ values in the population grouping pattern: [Shaanxi, Henan, Gansu,] [Qinghai, Sichuan, Yunnan, Hebei, Shanxi][Jilin, Liaoning, Heilongjiang]. In addition, there was significant differentiation for LSU sequences among sclerotia in the grouping pattern [Northwest China (Shaanxi, Gansu, Qinghai)][South China (Sichuan, Yunnan)][North China (Hebei, Shanxi)][Central Chain (Henan)][Northeast China (Jilin, Liaoning, Heilongjiang)] (Table 3).

**Table 2:** Pairwise population difference for ITS and LSU sequences of *Polyporus umbellatus* sclerotia.

	Hebei	Shanxi	Yunnan	Shaanxi	Henan	Gansu	Qinghai	Sichuan	Liaoning	Jilin	Heilongjiang
Hebei	0	3.50**	3	18.69	4	2.33	55	4	56	57	57.00*
	0	1.50*	9	9	12.2	3.00**	18.00*	3	23.00*	21	21.33*
Shanxi	2	3	4.5	18.88	3.8	2.5	54.50**	3.5	55.5	56.00*	56.00**
	1.17	0.66	8.5	8.8	11.5	2.5	17.50*	2.5	22.50*	20.5	20.83*
Yunnan	3	3	0	20.15*	5.4	3.33	55	1	57	58	58
	9	8.17	0	16.31**	19.2	10	25	10	30	28	28.33
Shaanxi	4.15	2.84	5.62	29.08	19.89*	18.1	66.31**	19.38	62.92*	63.92**	63.92**
	2.31	1.78	9.62	13.38	18.03**	10.31	21.62**	10.31	26.62	24.62*	24.95**
Henan	1.8	0.1	3.2	3.15	4.4	3.67	55.80**	4.8	56.8	57	57.00**
	3.9	2.87	10.9	3.04	16.6	12.93	22.60*	13.2	17.6	14.8	16.73
Gansu	1.33	0	2.33	2.56	0.46	2	52.67	3	54.33	55.33	55.33
	1.33	0.5	8.33	1.95	2.97	3.33	17.67	4	23.33	21.33	21.67
Qinghai	55	53	55	51.76	53.6	51.67	0	54	60	61	61
	18	17.17	25	14.92	14.3	16	0	19	25	23	23.33
Sichuan	4	2	1	4.84	2.6	2	54	0	56	57	57
	3	2.17	10	3.62	4.9	2.33	19	0	24	22	22.33
Liaoning	56	54	57	48.38	54.6	53.33	60	56	0	1	1
	23	22.17	30	19.92	9.3	21.67	25	24	0	4	6.33
Jilin	57	54.5	58	49.38	54.8	54.33	61	57	1	0	0
	21	20.17	28	17.92	6.5	19.67	23	22	4	0	4.33
Heilongjiang	57	54.5	58	49.38	54.8	54.33	61	57	1	0	0
	19.67	18.83	26.67	16.59	6.77	18.33	21.67	20.67	4.67	2.67	3.33

Above diagonal: Average number of pairwise differences between populations (PiXY); Diagonal elements: Average number of pairwise differences within population (PiX); Below diagonal: Corrected average pairwise difference (PiXY−(PiX+PiY)/2); P values of PXY are indicated (* p<0.05; **p<0.01).

**Table 3:** AMOVA for grouping of sclerotial populations using F-statistics based on ITS and LSU sequences.

Groups	Among populations within groups (F_SC_)	Within populations (F_ST_)	Among groups (F_CT_)
[Shaanxi, Gansu, Qinghai][Sichuan, Yunnan][Henan][Hebei, Shanxi][Jilin, Liaoning, Heilongjiang]	0.48** (0.35**)	0.67** (0.55**)	0.36* (0.30**)
[Shaanxi, Henan, Gansu] [Qinghai, Sichuan, Yunnan, Hebei, Shanxi][Jilin, Liaoning, Heilongjiang]	0.39** (0.37**)	0.69** (0.56**)	0.50** (0.30**)
[Shaanxi] [Henan, Qinghai, Sichuan, Yunnan, Gansu, Hebei, Shanxi, Jilin, Liaoning, Heilongjiang]	0.69** (0.56**)	0.59** (0.48**)	−0.31 (−0.19)
[Shaanxi, Henan] [Qinghai, Sichuan, Yunnan, Gansu, Hebei, Shanxi, Jilin, Liaoning, Heilongjiang]	0.65** (0.56**)	0.64** (0.50**)	−0.04 (−0.15)
[Shaanxi, Gansu, Qinghai, Hebei, Shanxi, Jilin, Liaoning, Heilongjiang] [Henan] [Sichuan, Yunnan]	0.68** (0.55**)	0.57** (0.47**)	−0.33 (−0.16)

Notes: *F*_SC_ estimates the variation among populations relative to a regional grouping of populations. *F*_ST_ estimates the proportions of genetic variation within populations relative to the genetic variation for the whole samples. *F*_CT_ estimates the proportion of genetic variation among groups of populations relative to the whole species. *F*_CT_ values that are statistically different are indicted. The P values are indicated as * <0.05, ** <0.01.

### ITS and LSU heterogeneity by PCR-SSCP analyses

**Figure 2:**
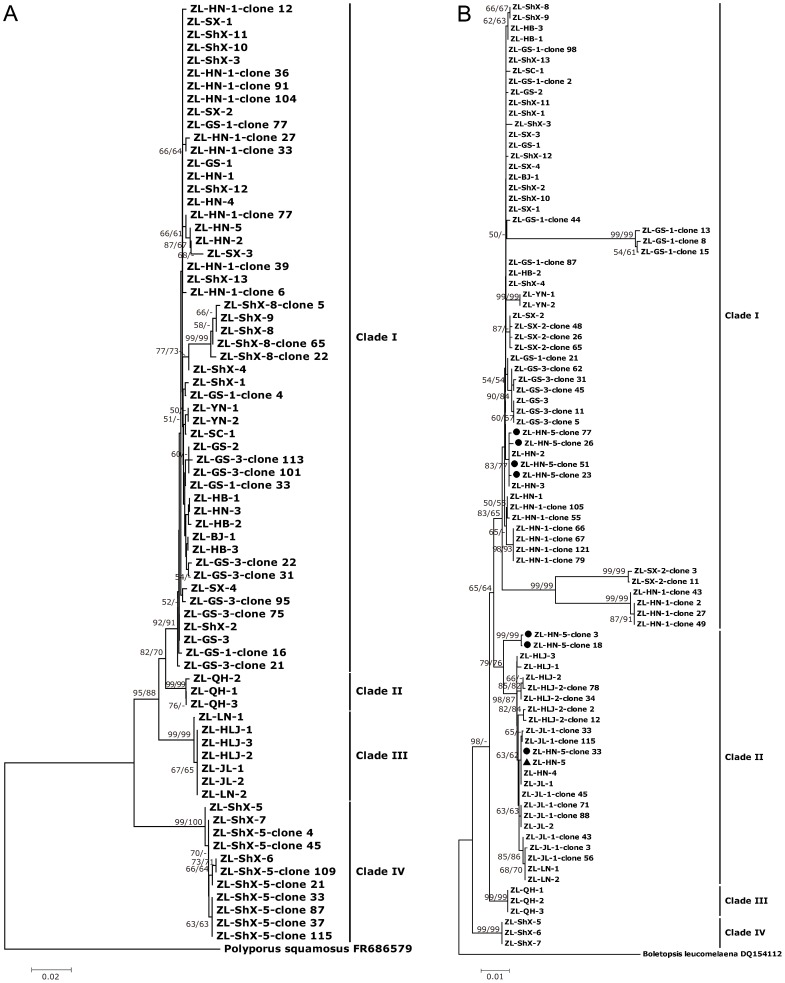
Phylogenetic analyses of ITS and LSU clones and their original sequences from direct sequencing. Bootstrap values from minimum evolution and maximum parsimony higher than 50% are shown at the nodes. 2A. Phylogenetic analyses based on ITS sequences of 31 clones and their original sequences from direct sequencing. *Polyporus squamosus* FR686579 was used as outgroup. The Arabic number after the clone represents clone number. 2B. Phylogenetic analyses based on LSU sequences of 49 clones and their original sequences from direct sequencing. *Boletopsis leucomelaena* DQ154112 was used as outgroup. “▴” indicates original sequence of ZL-HN-5. “•” indicates clones of ZL-HN-5. Samples represent different provinces (see Fig. 1 for legend).

In total, 577 ITS clones (from 5 samples: ZL-ShX-5, ZL-ShX-8, ZL-HN-1, ZL-GS-1, ZL-GS-3) and 683 LSU clones (from 6 samples: ZL-HN-1, ZL-HN-5, ZL-GS-1, ZL-GS-3, ZL-SX-2, ZL-JL-1 and ZL-HLJ-2) were analyzed by SSCP. Thirty-one ITS clones and 49 LSU clones with different SSCP patterns were sequenced. Compared with the corresponding original sequence (from direct sequencing), ITS heterogeneity was detected for clones from 1 sample (ZL-ShX-5) and LSU heterogeneity was detected in 4 samples (ZL-SX-2, ZL-JL-1, ZL-HLJ-2 and ZL-HN-5) Both ITS and LSU heterogeneity was detected in clones from 3 samples (ZL-HN-1, ZL-GS-1 and ZL-GS-3) (Table 4). For ITS, all clone sequences clustered with the clade identified using direct sequencing (Fig. 2A). For LSU, clones from the 7 samples clustered in agreement with direct sequencing except for ZL-HN-5. Twenty-three informative sites were observed among 7 clones of ZL-HN-5. Among these 23 informative sites, 20 changes occurred at known informative sites and one at known singleton sites. The possible nucleotide change at these informative sites was shared by at least two clones. Interestingly, the 7 clones of ZL-HN-5 were scattered between two clades (clade I and clade II)(Fig. 2B).

**Table 4:** PCR-SSCP results of ITS and LSU sequences of *Polyporus umbellatus*.

	ITS sequences				LSU sequences			
	No. of tested clones	No. of sequenced clones	No. of polymorphic sites^a^	Nucleotide diversity, Pi (%)	No. of tested clones	No. of sequenced clones	No. of polymorphic sites^a^	Nucleotide diversity, Pi (%)
ZL-ShX-5	128	8	5 (3)	0.29±0.12	–	–	–	–
ZL-ShX-8	67	3	0 (4)	0.43±0. 21	–	–	–	–
ZL-HN-1	156	9	4 (2)	0.20±0.13	121	10	61 (1)	2.28±0.20
ZL-HN-5	–	–	–	–	93	7	23 (7)	1.01±0.16
ZL-GS-1	95	4	2 (2)	0.27±0.15	98	8	68 (3)	2.62±0.23
ZL-GS-3	131	7	4 (2)	0.42±0. 16	78	5	2 (1)	0.11±0.01
ZL-SX-2	–	–	–	–	83	5	58 (5)	2.60±0.27
ZL-JL-1	–	–	–	–	122	8	5 (1)	0.18±0.07
ZL-HLJ-2	–	–	–	–	88	6	5 (4)	0.38±0.12
Total	577	31	–	–	683	49	–	–

a Numbers outside parentheses are parsimony informative sites, inside parentheses are singleton variable sites.

## Discussion

### Variation in ITS rDNA and LSU

We found evidence of four clearly defined intraspecific clades of *P. umbellatus* based on a combination of multiple phylogenetic analyses and looking at two nuclear DNA regions (ITS rDNA and LSU). ITS sequences often vary within isolates of a single genus and in some cases in isolates of a single species [22]. The degree of intraspecific variation in the ITS region can vary considerably in different fungi [32], [33]. In our study, both the ITS and LSU varied significantly between different geographic regions in China. Four clades could be clearly defined based on ITS, LSU and their combined data. Three sclerotial samples from Hanyin, Shaanxi province (ZL-ShX-5, ZL-ShX-6 and ZL-ShX-7) had relatively distant genetic relationships from other samples. We also found ITS and LSU heterogeneity within some samples but the potential sources of heterogeneity are still unknown. Most of the clones clustered with the same clade as their original sequence (from direct sequencing). For ZL-HN-5 (Luanchuan, Henan Province) LSU, four LSU clones were clustered in another clade, suggesting that recombination might have occurred between different *P. umbellatus* populations.

Because rDNA is multicopy and fungi can be multinucleate, the different ITS and LSU sequences within one sclerotium may be due to the differences among ribosomal DNA copies within the same nucleus, or due to variation among different nuclei [34], since for *P. umbellatus*, the sclerotia are composed of closely interwoven dikaryotic mycelium (secondary mycelium). The relative contribution of these two possible mechanisms for intraspecific variation requires further study using monokaryotic cultures of *P. umbellatus*.

### Sclerotial morphology and genetic group

Our research rejected a genetic basis for the two different sclerotial morphotypes (*Zhu Shi Ling* and *Ji Shi Ling*). The only previous analysis on intraspecific genetic variation of *P. umbellatus* was on *Zhu Shi Ling* and *Ji Shi Ling* from same geographic site, and no genetic variation was found [13]. Contrary to previous findings, we found genetic variation within morphotypes. In our study, most of the samples were *Zhu Shi Ling* except for samples from Hanyin, Shaanxi Province and from Northeast China (Jilin, Liaoning and Heilongjiang) which were *Ji Shi Ling*. Interestingly, these *Ji Shi Ling* samples formed two distinct clades in the phylogenetic trees. A similar result was also for *Zhu Shi Ling* samples from Qinghai Province, which formed a separate clade compared with other *Zhu Shi Ling* samples. These results indicate that sclerotia presenting the same morphology may belong to different phylotypes and that sclerotial morphology seems to be influenced by factors other than, or in addition to, genetics.

### Origin and evolution of *P. umbellatus*

In addition to detecting variation among populations, the ITS/LSU region has also been used to answer questions about gene flow between populations and the issues of allopatric speciation as result of geographic separation [35]. In this study, significant genetic divergence within the species of *P. umbellatus* from different geographic regions was revealed by two genes (nrDNA ITS and LSU) and four groups were detected. The considerable base changes indicated that recombination might have occurred between different *P. umbellatus* populations. Samples from Shaanxi and Henan province were of the highest nucleotide diversity of ITS and LSU. Additionally, significant genetic differentiation was observed between Shaanxi, Gansu, Henan and other populations. However, because *P. umbellatus* is so rare in some provinces, small sample size may have led to an underestimation of genetic diversity of *P. umbellatus*. Although there was discrepancy among sample size in this study, as revealed by our data, Shaanxi has greater within-populations genetic diversity than other populations. SSCP analysis revealed that ITS or LSU heterogeneity occurred in nine samples (sclerotia from Shaanxi, Henan, Gansu, Shanxi, Jilin and Heilongjiang), both ITS and LSU heterogeneity was only detected in samples from Henan and Gansu. These results support the idea that Shaanxi, Henan and Gansu may act as one of centers of origin of *P. umbellatus*. The westward and southward spread may have initiated along two parallel mountain ranges, Qinling Mountain and Daba Mountain, while the northward and eastward spread might have been associated with the differentiation of the Taihang Mountain and Changbai mountain range.

Although we know little about human-mediated dispersal of *P. umbellatus*, there is evidence for the use of *P. umbellatus* sclerotia 2000 years ago. The use of *P. umbellatus* sclerotia in Chinese medicine was first recorded in “*Shen Nong Ben Cao Jing* (Shen Nong's classic of the Materia Medica)”, the earliest book on material medic (circa 101BC) [36]. Thus it is possible that the current distribution of *P. umbellatus* was influenced, in part, by human activities but this requires further study.

### Genetic structure and medicinal value of *P. umbellatus*

Based on molecular evidence, we found evidence for four groups of *P. umbellatus*. The population structure analyses suggested that gene flow and population structure existed among sclerotia of *P. umbellatus*. In order to clarify whether the four groups of *P. umbellatus* are correlated with other phenotypic characters such as finer morphological differences in basidia and basidiospores, further study is required. Certainly, these differences might extend to variation in bioactive compounds. Except for the early reported sclerotial polysaccharide [37], other bioactive compounds such as diuretic compounds [38], [39], anti-inflammatory ergostane-type ecdysteroids etc. [40] have been reported in recent years. Information about the genetic structure of *P. umbellatus* combined with its medical value will help inform the cultivation and conservation of this rare fungus.

In conclusion, we showed strong support for different genetic lineages within a wide geographic area for *P. umbellatus*. Based on our evidence, it will be important to determine which of these lineages produces sclerotia most appropriate for pharmaceutical use.

## Supporting Information

Table S1 **Sclerotia of** ***Polyporus umbellatus*** **used in this study.** For each population, natural sclerotia were collected from mountains and usually came from different towns or counties. Sequences length of nrDNA ITS and 28S rRNA (LSU) for each sample are shown.
(XLS)

Table S2 **Sequence differences among seven haplotypes found in the nrDNA ITS region.**
(XLS)

Table S3 **Sequence differences among eight haplotypes found in the LSU region.**
(XLS)

Figure S1 **Phylogenetic analyses of 42** ***Polyporus umbellatus*** **sclerotia based on ITS sequences (A) and LSU sequences (B).** Bootstrap values from minimum evolution, maximum parsimony and maximum likelihood higher than 50% are shown at the nodes.
(TIF)

## References

[1] Lee JY (1988) Colored Korean mushrooms. Academy Press, Seoul.

[2] Imazeki R, Hongo T (1989) Colored illustrations of mushrooms of Japan. Vol. 2. Osaka, Japan: Hoikusha.

[3] Núnez M, Ryvarden L (1995) *Polyporus* (Basidiomycotina) and related genera. Synopsis Fungorum 10. Oslo, Norway: Fungiflora.

[4] Dai YC (2012) Polypore diversity in China with an annotated checklist of Chinese polypores. Mycoscience 53: 49–80.

[5] Xu JT (1997) Medicinal Mycology in China. Beijing, Beijing Medical University and Peking Union medical University Union Press

[6] Dai YC, Zhou LW, Yang ZL, Wen HA, Bao T, et al (2010) A revised checklist of edible fungi in China. Mycosystema 29: 1–21 (in Chinese).

[7] Dai YC, Yang ZL, Cui BK, Yu CJ, Zhou LW (2009) Species diversity and utilization of medicinal mushrooms and fungi in China. Int J Med Mushrooms 11: 287–302.

[8] The State Pharmacopoeia Commission of PR China (2010) Pharmacopoeia of the People's Republic of China, vol 1. Beijing, Chinese Medical Science and Technology Press

[9] Yang L, Wang R, Liu J, Tong H, Deng Y, et al (2004) The effect of *Polyporus umbellatus* polysaccharide on the immunosuppression property of culture supernatant of S180 cells. Chinese J Cell Mol Immunol 20: 234–237 (in Chinese). 15191734

[10] Zeng X, Li CX, Huang Y, Zhang GW, Zhang X, et al (2011) Effects of *Polyporus umbellatus* and *Polyporus* polysaccharide on the phagocytosis function and costimulatory molecules expression of peritoneal macrophages in rat bladder cancer. Chinese J Immunol 27: 414–418 (in Chinese).

[11] Li SQ (2008) Endangered *Polyporus umbellatus* resource needing protection and development-an investigation from producing areas. Modern Chinese Medicine 10 (6) 43–44 (in Chinese).

[12] Yao L, Cheng H, Yang Z (2006) Guidelines for good agricultural practice of Chinese crude drugs. China Agricultural Press, Beijing. (in Chinese)

[13] Liu K, Deng B, Chen W, Ding X, Peng H, et al (2009) Genetic relationships between three *Polyporus umbellatus* isolates based on DNA sequence analysis. Acta Edulis Fungi 16 (3) 11–14 (in Chinese).

[14] Dai YC (1996) Changbai wood-rotting fungi 5. Study on *Polyporus mongolicus* and *P. tubaeformis* . Ann Bot Fennici 33: 153–163.

[15] Sotome K, Hattori T, Ota Y, To-anun C, Salleh B, et al (2008) Phylogenetic relationships of *Polyporus* and morphologically allied genera. Mycologia 100: 603–615. 18833753 10.3852/07-191r

[16] Shen Q, Geiser DM, Royse DJ (2002) Molecular phylogenetic analysis of *Grifola frondosa* (maitake) reveals a species partition separating eastern North American and Asian isolates. Mycologia 94: 472–482. 21156518

[17] Ko KS, Jung HS (2002) Phylogenetic evaluation of *Polyporus s.str*. based on molecular sequences. Mycotaxon 82: 315–322.

[18] Krüger D, Gargas A (2004) The basidiomycete genus *Polyporus*-an emendation based on phylogeny and putative secondary structure of ribosomal RNA molecules. Feddes Repertorium 115: 530–546.

[19] Hibbett DS (1992) Ribosomal RNA and fungal systematics. Trans Mycol Soc Japan 33: 533–556.

[20] Binder M, Hibbett DS (2002) Higher-level phylogenetic relationships of homobasidiomycetes (mushroom-forming fungi) inferred from four rDNA regions. Mol Phylogenet Evol 22: 76–90. 11796031 10.1006/mpev.2001.1043

[21] Miettinen O, Larsson E, Sjokvist E, Larsson KH (2012) Comprehensive taxon sampling reveals unaccounted diversity and morphological plasticity in a group of dimitic polypores (Polyporales, Basidiomycota). Cladistics 28: 251–270. 10.1111/j.1096-0031.2011.00380.x 34872189

[22] White TJ, Bruns T, Lee S, Taylor J (1990) Amplification and direct sequencing of fungal ribosomal RNA genes for phylogenetics. In: Innis MA, Gelfand DH, Sninsky JJ, White TJ, eds. RCR protocols: a guide to methods and applications. New York, USA Academic Press

[23] Vilgalys R, Hester M (1990) Rapid genetic identification and mapping of enzymatically amplified ribosomal DNA from several species of Cryptococcus. J Bacteriol 172: 4238–4246. 2376561 10.1128/jb.172.8.4238-4246.1990 PMC213247

[24] Larkin MA, Blackshields G, Brown NP, Chenna R, McGettigan PA, et al (2007) Clustal W and Clustal X version 2.0. Bioinformatics 23: 2947–2948. 17846036 10.1093/bioinformatics/btm404

[25] Tamura K, Peterson D, Peterson N, Stecher G, Nei M, et al (2011) MEGA5: Molecular Evolutionary Genetics Analysis using Maximum Likelihood, Evolutionary Distance, and Maximum Parsimony Methods. Mol Biol Evol 28: 2731–2739. 21546353 10.1093/molbev/msr121 PMC3203626

[26] Darriba D, Taboada GL, Doallo R, Posada D (2012) jModelTest 2: more models, new heuristics and parallel computing. Nature Methods 9: 772. 10.1038/nmeth.2109 PMC4594756 22847109

[27] Swofford DL (2003) PAUP*: phylogenetic analysis using parsimony (*and other methods). Version 4.0b10. Massachusetts, USA: Sinauer Associates.

[28] Librado P, Rozas J (2009) DnaSP v5: A software for comprehensive analysis of DNA polymorphism data. Bioinformatics 25: 1451–1452. 19346325 10.1093/bioinformatics/btp187

[29] Excoffier L, Lischer HEL (2010) Arlequin suite ver 3.5: a new series of programs to perform population genetics analyses under Linux and Windows. Mol Ecol Resour 10: 564–567. 21565059 10.1111/j.1755-0998.2010.02847.x

[30] Sunnucks P, Wilson ACC, Beheregaray LB, Zenger K, French J, et al (2001) SSCP is not so difficult: the application and utility of single-stranded conformation polymorphism in evolutionary biology and molecular ecology. Molecul Ecol 9: 1699–1710. 10.1046/j.1365-294x.2000.01084.x 11091307

[31] Wang QM, Li J, Wang SA, Bai FY (2008) Rapid differentiation of phenotypically similar yeast species by single-strand conformation polymorphism of ribosomal DNA. Appl Environ Microbiol 74: 2604–2611. 18344345 10.1128/AEM.02223-07 PMC2394880

[32] Vasaitis R, Menkis A, Lim YW, Seok S, Tomsovsky M, et al (2009) Genetic variation and relationships in *Laetiporus sulphureus* s. lat., as determined by ITS rDNA sequences and in vitro growth rate. Mycol Res 113: 326–336. 19073254 10.1016/j.mycres.2008.11.009

[33] Pannecoucque J, Höfte M (2009) Detection of rDNA ITS polymorphism in *Rhizoctonia solani* AG 2-1 isolates. Mycologia 101: 26–33. 19271668 10.3852/08-084

[34] Okabe I, Matsumoto N (2003) Phylogenetic relationship of *Sclerotium rolfsii* (teleomorph *Athelia rolfsii*) and *S. delphinii* based on ITS sequences. Mycol Res 107: 164–168. 12747327 10.1017/s0953756203007160

[35] Hughes KW, McGhee LL, Methven AS, Johnson JE, Petersen RH (1999) Patterns of geographic speciation in the genus *Flammulina* based on sequences of the ribosomal ITS1-5.8S-ITS2 area. Mycologia 91: 978–986.

[36] Liu Z, Liu Y (2001) State of herbal medicines in China. J Herb Pharmacother 1: 25–34.

[37] Miyazaki T, Oikawa N (1973) Studies on fungal polysaccharide. XII. Water-soluble polysaccharide of *Grifora umbellata* (Fr.). Chem Pharm Bul 21: 2545–2548.

[38] Yuan D, Mori J, Komatsu KI, Makino T, Kano Y (2004) An anti-aldosteronic diuretic component in *Polyporus sclerotium* . Biol Pharm Bull 27: 867–870. 15187435 10.1248/bpb.27.867

[39] Zhao YY, Xie RM, Chao X, Zhang YM, Lin RC, et al (2009) Bioactivity-directed isolation, identification of diuretic compounds from *Polyporus umbellatus* . J Ethnopharmacol 126: 184–187. 19665537 10.1016/j.jep.2009.07.033

[40] Sun Y, Yasukawa K (2008) New anti-inflammatory ergostane-type ecdysteroids from the sclerotium of *Polyporus umbellatus* . Bioorgan Med Chem Lett 18: 3417–3420. 10.1016/j.bmcl.2008.04.008 18439824

