# Supplementary figures and images for: Genetic Diversity and Evolution of Chinese Traditional Medicinal Fungus Polyporus umbellatus (Polyporales, Basidiomycota)
Source: PLoS One. 2013 Mar 15;8(3):e58807. doi: 10.1371/journal.pone.0058807 (PMC3598919; doi:10.1371/journal.pone.0058807)

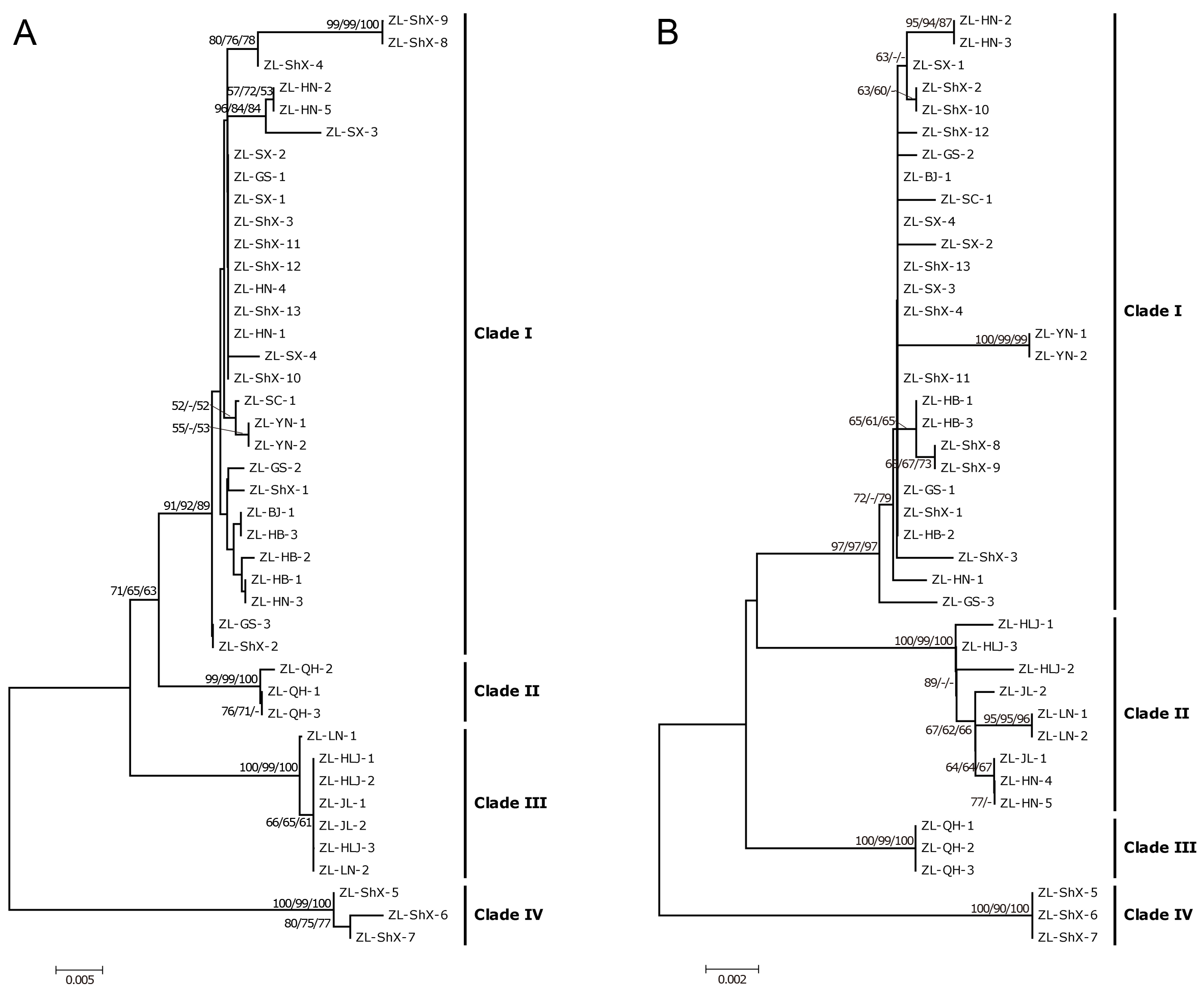

Supplement: Figure S1 — Phylogenetic analyses of 42 Polyporus umbellatus sclerotia based on ITS sequences (A) and LSU sequences (B). Bootstrap values from minimum evolution, maximum parsimony and maximum likelihood higher than 50% are shown at the nodes. (TIF) [file pone.0058807.s004.tif]
